# Assessing the students' readiness for E-Learning during the Covid-19 pandemic: A case study^☆^

**DOI:** 10.1016/j.heliyon.2022.e10219

**Published:** 2022-08-17

**Authors:** Habibollah Dehghan, Sayed Vahid Esmaeili, Fatemeh Paridokht, Nima Javadzade, Mehdi Jalali

**Affiliations:** a Department of Occupational Health and Safety Engineering, Faculty of Health, Isfahan University of Medical Sciences, Isfahan, Iran; b Occupational Health and Safety Engineering, Student Research Committee, Faculty of Health, Isfahan University of Medical Science, Isfahan, Iran; c Health Psychology, Student Research Committee, Faculty of Medicine, Isfahan University of Medical Sciences, Iran; d Department of Occupational Health Engineering, School of Health, Neyshabur University of Medical Sciences, Neyshabur, Iran

**Keywords:** E-Learning, Motivation, Readiness for E-learning, Virtual education

## Abstract

The aim of this study was to scrutinize the E-learning readiness among the students of the Faculty of Health at Isfahan University of Medical Sciences during the Covid-19 pandemic. This study was conducted as a descriptive-analytic study on 165 the students of the Faculty of Health in Isfahan University of Medical Sciences in 2021. To examine the level of the participants' readiness for E-learning during the Covid-19 pandemic period, the questionnaire developed by Watkins et al, was administered. Finally, the data analysis was performed with SPSS_24_ software. The results showed that the mean and standard deviation were 97.44 and 17.67, respectively. Only 1.2% of the participants had a low level of E-learning readiness. 27.9% were at the intermediate level and 70.9% were at the high level. Moreover, the results revealed that the participants had the highest degree of readiness in "Technology Access" and "Internet discussions" dimensions (0.79) and the lowest degree of readiness in "motivation" dimension (0.67). The results of this study indicated that the majority of the participants in this study had a high E-learning readiness, so the university can implement the virtual education. However, the students' learning motivation should be enhanced through providing the required infrastructure and holding training courses for the students specially the BA ones.

## 1 Introduction

By the end of 2019, Covid-19 disease began to spread worldwide [1]. This disease affected not only the health systems but also the other fields including education. Due to the Covid-19 increased prevalence, and the high probability of being infected while communicating in educational settings, the existence of physical distance among people has been highly emphasized [2, 3, 4]. Accordingly, in-person training in schools and universities was cancelled in many countries including Iran to decrease the Covid-19 prevalence [3]. In many parts of the world, educational services were totally provided virtually [5] which in turn made 1.5 billion learners deprived of in-person training in early 2020 [6].

Virtual learning refers to a learning environment in which teachers and learners are separate from each other in terms of either time or place or both [7]. In other words, electronic learning is done through the use of electronic training systems such as, computers, multi-purpose CDs, internet, electronic journals and newsletters [8]. On the other hand, E-learning readiness points to people's ability to benefit from electronic and multi-media technological training resources to enhance the learning quality [9].

The advantages of E-learning are "being at home", "reducing costs", "using electronic devices in different times and places" and "being flexible in starting and ending the sessions" [10]. Besides, "easy access to the resources", "session recording", "on-going updating" and "24-h availability" are the other advantages [11]. However, E-learning has several disadvantages such as "high costs of launching computer systems and devices", "high costs of using telephone lines", "inaccessibility of computers for all people", "schools' unawareness of the copyright laws", "Internet accessibility problems", "low quality of Internet connections", and "respondents' inadequate computer skills" [11, 12].

Despite the fact that the demand for E-learning opportunities has increased in recent years, many professionals are beginning to question whether students are prepared to succeed in an E-learning environment [13]. In the same line, several studies examined the challenges attributed to E-learning readiness. The studies evidenced that implementing E-learning innovations failed due to the universities and educational institutes' lack of preparation. An investigation titled "The students' perceptions of E-learning strategies during Covid-19 pandemic" indicated that the change of in-person training to virtual training resulted in the students' academic decline [14]. Another study conducted in Saudi Arabia during Covid-19 pandemic revealed that virtual learning is not always attractive [5]. Aguilera-hermida also showed that students prefer in-person training to virtual training. Further, the students believed that virtual learning was more difficult and the lack of support resources was an important problem during E-learning evolution [15]. However, virtual medical education including clinically simulated scenarios prevented training discontinuation and most the students reacted positively toward the perceived quality of this method. But, the number of the students who had trouble accessing on-line was not negligible [16]. But a study by, Michael Co and Kent-Man Chu found that students who complete face-to-face and web-based teaching are more likely to find web-based online teaching a viable alternative to face-to-face teaching [17]. Also, Dalili Saleh et al. evidenced that E-learning development among the students of Sabzevar University of Medical Sciences was accompanied by the students' lack of readiness [18].

After all, the students' reported success in traditional education may not be a good predictor of their success in an E-learning environment [19, 20]. Students should have technical training and abilities in working with the related devices because such learning environments require a technology-mediated setting. This has raised the question of whether the higher education students in Iran are adequately equipped for E-learning and ready to cope with the associated challenges. Hence, this study was undertaken to assess the E-learning readiness of the students of the Health Faculty at Isfahan University of Medical Sciences during COVID-19 and its association with demographic variables. The results are hoped to recognize the E-learning points of strength and weakness in Iranian academic context.

## 2 Method

### 2.1 Participants

The present study, being cross-sectional (descriptive-analytic) in design, aimed to examine the degree of E-learning readiness among the students of the Health Faculty at Isfahan University of Medical Sciences during Covid-19 pandemic in 2020. This study was approved by the ethics committee of Isfahan University of Medical Sciences with reference number of IR. MUI.RESEARCH.REC.1400.006. All participants completed and signed the consent form to participate in the study. The sample size was determined using the Krejcie and Morgan table. As the study population was 290 students, the maximum sample size was calculated to be 165 students. The participants were selected through stratified random sampling method from three subgroups of bachelor, M.Sc. and Ph.D. As to the selection criterion, the selected students were those who had attended virtual classrooms for at least one semester during Covid-19 Pandemic.

### 2.2 Instrument and data collection

The instrument used in this study was the Watkins et al.'s standard E-learning readiness questionnaire [21]. This questionnaire included 26 items examining the students' perceptions about their readiness for E-learning in terms of six factors (Technology Access, Online Skills and Relationships, Motivation, Online Audio/Video, Internet Discussions, Importance to your success). The questionnaire responses were measured based on a 5-point Likert scale (strongly disagree = 1, disagree = 2, no idea = 3, agree = 4, and strongly agree = 5) [9]. The higher the scores were, the readier the students were for E-learning. Therefore, the lowest possible score was 26 and the highest one was 130. Further, the obtained scores could be categorized into three levels of "low" (below 45), "intermediate" (between 45 and 90), and "high" (above 90) [22]. The reliability of the Persian version of the questionnaire had been measured by using Cronbach's alpha in the Hedayati study (r = 0.92) [23]. The participants' demographic information (age, gender, educational level, marital status, employment status) was collected using a checklist. The web link of the questionnaire and the checklist, designed by Porsline software were sent to the participants through social networks (such as: WhatsApp, Telegram, etc.). The link also included the consent form the participants were supposed to fill out.

### 2.3 Data analysis

SPSS Version 24 was used for the data analysis. The data were descriptively analysed using frequency and percentages. In order to compare the means of E-learning scores in each group pairs (male and female/married and single/employed and unemployed), one sample t-test was used. A one-way ANOVA test was used to compare the means of E-learning scores among the three groups of bachelor, M.Sc. and Ph.D. All tests were conducted at a 95% significance level with α = 5%.

## 3 Results

The present study was conducted on 165 students of the Health Faculty at Isfahan University of Medical Sciences during Covid-19 pandemic. The questionnaire was completed by 165 out of 231 (71.4%) students to whom it was sent. The mean and standard deviation of the participants' age were 25.5 and 5.33, respectively, with 18 as the minimum and 40 as the maximum. Table 1 reports the descriptive results of the participants' demographic characteristics. 48 males (29.1%) and 117 females (70.9%) participated in this study.

**Table 1:** The participants' demographic characteristics.

Variable		Frequency	Percentage
Gender	Male	48	29.1
	Female	117	70.9
Marital Status	**Single**	139	84.2
	**Married**	26	15.8
Level Of Education	**Ba**	80	48.5
	**M.Sc**	55	33.3
	**Ph.D.**	30	18.2
Employment Status	**Unemployed**	103	62.4
	**Employed**	62	37.6

Table 2 indicates the descriptive results of the participants' perceptions of their readiness for E-learning. The results revealed that the mean and the standard deviation of the responses were 97.44 and 17.67, respectively. The ratio of the mean score to the highest possible score in each factor showed that the participants rated the Technology Access (0.79) and Internet Discussions (0.79) factors as the highest and the Motivation (0.67) factor as the lowest. However, the proximity of these ratios to each other pointed to the fact that the participants' perceptions about all the factors were almost the same. As to the E-learning readiness levels, the results indicated that only 1.2% of the participants were at the low readiness level. Interestingly, 27.9% of the participants were at the intermediate and 70.9% at high readiness levels.

**Table 2:** Descriptive results of the participants' perceptions of their E-learning readiness.

Readiness factor	N∗	Mean (M)	SD	Minimum	Maximum	The highest possible score (A)	M/A∗∗
Online skills and relationships	9	34.7	6.76	9	45	45	0.77
Motivation	3	10.15	2.74	3	15	15	0.67
Technology Access	3	11.81	3.28	3	15	15	0.79
Online Audio/Video	3	11.67	2.65	3	15	15	0.78
Internet Discussions	3	11.84	2.31	3	15	15	0.79
Importance to your Success	5	17.23	4.09	9	15	25	0.69
Total	26	97.44	17.67	30	120	130	0.75

∗ the number of items for each factor.

∗∗ the ratio of the mean score to the highest possible score in each factor.

Table 3 reports the results of comparing the means of different groups of participants in terms of their demographic characteristics. T-test results for comparing the male and females' mean scores indicated that the differences were not statistically significant (P = 0.283). The same was true regarding the mean differences between the single and married participants (P = 0.489). However, the mean differences between employed and unemployed participants were statistically significant (P = 0.005) with employed participants having higher scores than the unemployed ones.

**Table 3:** The results of comparing the mean differences among the participants in terms of their demographic characteristics.

Variable		Mean	SD	P Value
Gender	Male	94.73	22.43	0.283
	Female	98.55	15.27	
Level Of Education	**Ba**	94.10	18.36	0.022
	**M.Sc**	98.60	13.27	
	**Ph.D.**	104.23	20.95	
Marital Status	**Married**	95.23	22.22	0.489
	**Single**	97.85	16.75	
Employment Status	**Employed**	102.08	13.94	0.005
	**Unemployed**	94.65	9.10	

Regarding the participants' educational levels, one-way ANOVA was run to see if the mean differences among the three groups were statistically significant. The results showed that there were statistically significant differences (P = 0.022) among them. Further, the results of performing post-hoc Tokay test indicated that the only statistically significant differences were between the BA and Ph.D. groups (P = 0.019) and the differences between the BA and M.Sc. and between M.Sc. and Ph.D. were not statistically significant (P = 0.304 and P = 0.329, respectively). As to the relationship between the participants' E-learning readiness scores and their age, the results of running Pearson test revealed a statistically significant relationship (R2 = 0.198, P = 0.011).

## 4 Discussion

The world-wide spread of Covid-19 made educational institutes turn to the virtual education [24]. However, in many countries, school and university students did not have identical resources for proper performance in the new conditions, which, in addition to the unpreparedness of e-learning, caused students to experience psychosocial impact related to e-learning [25, 26, 27]. The present study sought to investigate the degree of E-learning readiness among the students of the Health Faculty at Isfahan University of Medical Sciences during Covid-19 pandemic in 2020.

The obtained results showed that during Covid-19 pandemic 70% of the students had high degree of E-learning readiness. Most of the participants had access to the computers connected to the high-speed Internet and applications such as Microsoft Words, Adobe connect, and Skyroom. In contrast, Aboagye et. Al. found that the students did not have the required readiness for experiencing virtual education.

The results in the present study revealed that the most important challenge the students face was the low level of learning motivation. This may lend support to Rozhkova et. al. in that about 70% of the students do not have the required E-learning readiness due to the inadequacy of their motivation [28]. This may be accounted for by the absence of face-to-face communication between the students and their teachers, the lack of adequate supervision on the students' educational activities and the teachers' failure to use modern and updated teaching methods. Anwar et. al., found that virtual education resulted in the enhancement of the students' motivation [29].

The results also evidenced that the students rated the two factors of "Technology Access" and "Internet Discussions" the highest, so improving the technological facilities in this regard can enhance the students' readiness for E-learning. The lowest score was related to motivation which is in line with Ali-Asghari who identified 'access to technology' and 'motivation' as the two most effective factors in the scores [30]. Therefore, according to the current situation, if this educational system is established, the students' motivation to benefit from online classes and distance learning should be enhanced. Dalili et al.'s study also stated that providing students with high-speed Internet and with no network disruptions, as well as a quiet environment at home can enhance students' motivation [18]. This is in line with Ilvica Grace et. al., which found that the availability of technology was one of the challenges influencing students' E-learning during Covid-19 pandemic [31]. Also, Muilenburg and Berge identified the costs and Internet connection as the two less important barriers for virtual education [32]. These two factors, in fact, affect the accessibility to Modern technology which in turn has been recognized as an important factor in E-learning readiness in the present study.

The findings of this study also indicated that the females' mean score of E-learning readiness was higher than that of males, and that female students tend to be better prepared than male students. These findings are consistent with the results of a study by Almomani et al. According to the studies, female students' better satisfaction with the online learning experience is attributed to the value and interest that female students place in planning and participation, which can increase their readiness for E-learning [33]. Nevertheless, further analysis of the data as to the participants' marital status indicated there were no significant differences in the two groups' mean scores, which was in line with the previous studies [34, 35].

The results of comparing the means of the three groups with different educational levels showed that there were significant differences between BA and Ph. D groups. It can be inferred that the BA students are not adequately ready for E-learning. This may be explained by the high number of students in this level who have recently had fundamental changes in their educational settings and, therefore, are reluctant to accept any other change in their education. Another justification can be the sudden spread of Covid-19 which made this change fast and mandatory. Another reason for the difference in preparation between the BA group and two other groups can be due to the more use of computers and the Internet by students in higher education for their term projects, research studies and thesis [36]. This can be supported by Abbasi et. al., which found that BA student were more willing to have in-person education and not interested in virtual education [37].

Another variable under investigation in this study was the participants' employment status. The results indicated that the employed participants had higher degree of readiness for E-learning than the unemployed ones, even though the unemployed might have more free time to have access to virtual education. However, the employed students may be more familiar with the mechanisms of virtual education due to their job-related responsibilities. Regarding the relationship between the participants' perceptions about their readiness for E-learning and their age, there was a significant positive correlation, meaning that the higher the participants' age was, the higher their E-learning readiness was. This finding can be accounted for by the fact that the younger participants were probably BA students whose educational environment had just changed. This change, which was also coincided with the spread of Covid-19 and its consequences for the educational system, made it difficult for these students to accept the requirements of virtual education, supporting the results obtained by Hassan et. al [35].

Like any other studies, the current study suffers from certain limitations. Since this study is a cross-sectional one, the interpretation of the results should be limited for a certain period of time. Moreover, the data were collected from the participants in the peak of the disease, so the cognitive and affective factors might have influenced on their perceptions about their readiness for E-learning. Another variable not considered in the present study was the participants' residence, being urban or rural, which had probably made the participants different in terms of their accessibility to the technological devices and the quality of their Internet connections. Considering all these limitations, the results highlighted the urgent need to revisit the assessment of the present situation in terms of the requirements for running virtual education, improving the students' participation in on-line group discussions and communication, and enhancing their learning motivation. Future research should also discuss the effectiveness of online teaching approaches used for the students in different disciplines during the epidemic. Also, one of the factors influencing students' E-readiness can be the individual's financial status (socio-economic status), which should be considered in future research.

## 5 Conclusion

Some situations provide opportunities to learn new skills. The COVID-19 epidemic has made many students around the world adapt to the process of teaching and learning online. The results of the present study also showed that most of the participants were highly prepared for E-learning. So, the university can implement virtual education. However, it is necessary to increase the students' (especially BA students) motivation to learn by providing them with the required infrastructure and training courses to learn new skills and softwares.

## Declarations

### Author contribution statement

Habibollah Dehghan: Conceived and designed the experiments; Wrote the paper.

Nima Javadzade: Conceived and designed the experiments; Contributed reagents, materials, analysis tools or data; Wrote the paper.

Sayed Vahid Esmaeili & Fatemeh Paridokht: Performed the experiments; Wrote the paper.

Mahdi jalali: Analyzed and interpreted the data; Contributed reagents, materials, analysis tools or data.

### Funding statement

Nima Javadzade was supported by Isfahan University of Medical Sciences [199552].

### Data availability statement

Data included in article/supp. material/referenced in article.

### Declaration of interests statement

The authors declare no conflict of interest.

### Additional information

No additional information is available for this paper.
